# Cycling and Diabetes Prevention: Practice-Based Evidence for Public Health Action

**DOI:** 10.1371/journal.pmed.1002077

**Published:** 2016-07-12

**Authors:** Jenna Panter, David Ogilvie

**Affiliations:** MRC Epidemiology Unit and UKCRC Centre for Diet and Activity Research (CEDAR), University of Cambridge, Cambridge, United Kingdom

## Abstract

Panter and Ogilvie describe findings that link recreational and commuter cycling with reduced incidence of diabetes.

Epidemiological evidence indicates a strong protective effect of physical activity against morbidity and mortality from cardiovascular disease, diabetes, and some cancers [1]. Public health guidelines highlight the importance of regular activity, recommending at least 30 minutes of moderate-intensity activity on most days of the week for adults, and more for children [1]. However, levels of activity vary between countries, and a sizeable proportion of the global population would benefit from being more active [2]. While sport offers one route to health benefits for those who participate, active travel (walking and cycling) may offer an easier way for many people to integrate more exercise into their daily lives. A number of cohort studies have demonstrated a beneficial effect of active commuting on cardiovascular outcomes [3], and even after taking the hazards of air pollution and injuries into account, modelling suggests that a population shift toward more active travel would bring about substantial health gain and environmental co-benefits [4].

A study by Martin Rasmussen and colleagues published in this week’s *PLOS Medicine* examines the impact of cycling for commuting and recreation on the risk of developing type 2 diabetes, contributing valuable new evidence in this area [5]. In this cohort study of Danish adults recruited between the ages of 50 and 65, those who reported higher weekly quantities of cycling were less likely to develop diabetes, particularly in respect to cycling to and from work. Strengths of the study include its longitudinal design, large sample, and ascertainment of diabetes diagnoses from a national registry. Exposure to cycling was derived from a self-reported physical activity questionnaire, which performs well in terms of ranking participants’ physical activity levels. Future research in more recently established cohorts with more precise, objective measures of the behavioural exposures may contribute further to our understanding of these relationships in time.

Perhaps the most interesting and original finding of the study is that those who took up cycling after the study began also had a lower risk of developing diabetes than those who did not. This suggests that it is not too late to gain the benefits of taking up cycling, even in the years approaching retirement. In this study, participants in the group with the highest level of exposure reported an average of more than seven hours of cycling per week. In a recent study of the mortality benefits of cycling in a cohort of adults in England, by contrast, the average cyclist in the sample reported only about a third of this weekly quantity of cycling at baseline, and about a fifth at follow-up [6]. This begs the question of how other countries might shift their population distribution of cycling to a level sufficient to achieve the health benefits observed in this Danish study [7].

Although the authors mention the qualified evidence for the effectiveness of some individual behaviour change techniques, such as personalised travel planning [8], we have no reason to believe that those methods explain how countries like Denmark or the Netherlands have achieved and maintained their high prevalence of cycling. The key is more likely to involve the broader infrastructural strategies to which the authors also refer. A genuinely population-based public health strategy for the prevention of diabetes and other chronic diseases would address their fundamental social causes, seeking to change the circumstances in which people live and the environments and policies that shape those circumstances. In this arena of upstream, primordial prevention, interventions may not necessarily be focused on disease prevention at all. Instead, they may achieve that objective more obliquely by targeting leverage points in the systems that generate and sustain the behaviour patterns linked with chronic disease outcomes, such as the planning of towns and cities and the relative cost and convenience of different modes of transport [9].

Evidence for the health benefits of physical activity is now complemented by a growing set of observational studies linking physical activity with attributes of the physical environment [10]. This latter body of evidence suggests, for example, that if people either have to walk because they have no choice, or live in environments where there are places to walk to and it is comparatively direct, pleasant, and safe to do so, then they are more likely to walk more. While such epidemiological research provides increasing justification for efforts to change environments, the more pressing question for public health research is how to make it happen. The evaluation of primordial preventive strategies, such as urban planning and transport interventions, generally entails non-randomised study designs following a natural experimental paradigm—a challenging area of research in which interventions are not introduced for the purposes of evaluation and the researcher has no control over them [11]. No wonder, then, that it is more common to see papers calling for this type of research than to see papers reporting it. The evidence available to guide policy and practice in this area has long been subject to an evaluative bias in favour of interventions that are easier to evaluate, or perhaps easier to randomise [12]. But those are not necessarily the most effective public health strategies, so “it’s time to think smarter about the kind of research we need” [13].

The public health research community is now rising to this challenge, and more robust evidence for population-level interventions to shift activity patterns is beginning to emerge [14]. The future of diabetes prevention is likely to depend on adopting more ambitious, innovative, and radical public health actions, rather than merely continuing to apply existing “weak prevention” methods with greater intensity [9]. It is inevitable that some strategies will be more successful than others and that any given “solution” may generate new problems, but these should not be taken as reasons for inaction. The way forward will entail both professional and political leadership that is willing to take risks, as well as closer working between the research, policy, and practice communities to ensure that interventions can be rigorously evaluated, the findings disseminated, and effective strategies scaled up across the world [15].
